# AGE DIFFERENCES EMOTION GLOBALIZING: AN EXAMINATION OF BOUNDARY CONDITIONS

**DOI:** 10.1093/geroni/igac059.581

**Published:** 2022-12-20

**Authors:** Meaghan Barlow, Emily Willroth, Carsten Wrosch, Oliver John, Iris Mauss

**Affiliations:** Wilfrid Laurier University, Waterloo, Ontario, Canada; Washington University in St. Louis, St. Louis, Missouri, United States; Concordia University, Montreal, Quebec, Canada; UC Berkeley, Berkeley, California, United States; UC Berkeley, Berkeley, California, United States

## Abstract

Emotion globalizing is an individual difference variable referring to the extent to which daily variations in an individual’s current emotions spillover into their evaluations of life satisfaction. The present research sought to: 1) extend the conception of emotion globalizing to stressor-related emotions, 2) examine age differences in these processes, and 3) differentiate associations with evaluations of life satisfaction and day satisfaction. To do so, we used daily diary data from two adult lifespan community samples [Study 1: N = 133 females, age range = 23-78; Study 2: N = 137, age range = 18-95]. Multilevel models revealed older (compared to younger) adults exhibited less negative (but not positive) emotion globalizing and stressor-related emotion globalizing. No age differences were revealed in the association between stressor-related emotions and day satisfaction. These findings support and extend assumptions underlying the mechanisms of emotion globalizing and inform theories of emotional aging.

